# 3D analyses reveal T cells with activated nuclear features in T-cell/histiocyte-rich large B-cell lymphoma

**DOI:** 10.1038/s41379-022-01016-8

**Published:** 2022-02-16

**Authors:** Aresu Sadeghi Shoreh Deli, Sonja Scharf, Yvonne Steiner, Julia Bein, Martin-Leo Hansmann, Sylvia Hartmann

**Affiliations:** 1 Dr. Senckenberg Institute of Pathology, Goethe University Frankfurt, Theodor-Stern-Kai 7, D-60590 Frankfurt am Main, Germany; 2 Frankfurt Institute of Advanced Studies, Ruth-Moufang-Str. 1, 60438 Frankfurt am Main, Germany; 3 Molecular Bioinformatics, Goethe University Frankfurt am Main, Robert-Mayer-Str. 11-15, 60325 Frankfurt am Main, Germany; 4 Institute of General Pharmacology and Toxicology, Goethe University Frankfurt, Theodor-Stern-Kai 7, D-60590 Frankfurt am Main, Germany

**Keywords:** Hodgkin lymphoma, Preclinical research

## Abstract

Nodular lymphocyte-predominant Hodgkin lymphoma (NLPHL) can show variable histological growth patterns and present remarkable overlap with T-cell/histiocyte-rich large B-cell lymphoma (THRLBCL). Previous studies suggest that NLPHL histological variants represent progression forms of NLPHL and THRLBCL transformation in aggressive disease. Since molecular studies of both lymphomas are limited due to the low number of tumor cells, the present study aimed to learn if a better understanding of these lymphomas is possible via detailed measurements of nuclear and cell size features in 2D and 3D sections. Whereas no significant differences were visible in 2D analyses, a slightly increased nuclear volume and a significantly enlarged cell size were noted in 3D measurements of the tumor cells of THRLBCL in comparison to typical NLPHL cases. Interestingly, not only was the size of the tumor cells increased in THRLBCL but also the nuclear volume of concomitant T cells in the reactive infiltrate when compared with typical NLPHL. Particularly CD8^+^ T cells had frequent contacts to tumor cells of THRLBCL. However, the nuclear volume of B cells was comparable in all cases. These results clearly demonstrate that 3D tissue analyses are superior to conventional 2D analyses of histological sections. Furthermore, the results point to a strong activation of T cells in THRLBCL, representing a cytotoxic response against the tumor cells with unclear effectiveness, resulting in enhanced swelling of the tumor cell bodies and limiting proliferative potential. Further molecular studies combining 3D tissue analyses and molecular data will help to gain profound insight into these ill-defined cellular processes.

## Introduction

Nodular lymphocyte-predominant Hodgkin lymphoma (NLPHL) is a subtype of Hodgkin lymphoma that accounts for ~5–10% of all Hodgkin cases^1^. It is derived from germinal center B cells^2^. The tumor cells, i.e., the lymphocyte-predominant (LP) cells, have functional B-cell receptors and express B-cell antigens like CD20, CD79a, PAX5, and OCT2^3,4^. NLPHL mainly affects young-to-middle-aged males^5^. Most patients are diagnosed with early stages of the disease^6^. Different histopathological growth patterns have been observed in NLPHL (patterns A-F), described in detail by Fan et al.^7^. Since several histopathological growth patterns are frequently found within one lymph node, we proposed to divide cases into typical NLPHL (comprising patterns A and B according to Fan et al.^7^) and atypical variants comprising all other patterns. Atypical variants (patterns C-F according to Fan et al.^7^) were shown to manifest more frequently with advanced-stage disease and to have an increased risk of relapse when compared with typical nodular growth patterns^5^. Some cases of NLPHL, usually with Fan pattern C, show immunoglobulin heavy chain D (IgD) expression in the tumor cells^8^. We could recently show that these cases are triggered by infection with *M. catarrhalis*^9^. Restriction to particular major histocompatibility complex haplotypes points to an effect of cognate T-cell help in the development of NLPHL^9^. Additionally, we could show that the LP cells of NLPHL cases with patterns A-C interact with T cells rosetting around the LP cells in immunological synapses^10,11^. Some NLPHL cases with a variant histology (pattern E according to Fan et al.^7^) are difficult to differentiate from T-cell/histiocyte-rich large B-cell lymphoma (THRLBCL). Patients with THRLBCL are, like NLPHL patients, frequently middle-aged males (57–88%)^12,13^. However, in contrast to most NLPHL patients, they usually present with an advanced Ann Arbor stage and B-symptoms^13^. On the grounds of morphology, gene expression, and genetic data, an important overlap between cases of NLPHL Fan pattern E and THRLBCL has been observed. Data characterizing genomic aberrations as well as mutation analyses suggest progression from typical to variant patterns with a final transformation into THRLBCL^14,15^. However, since the tumor cell content in both NLPHL and THRLBCL is usually below 5% of the total infiltrate, global sequencing studies of the tumor cells are lacking so far. Since the shape and size of tumor nuclei frequently reflect the genomic complexity of a tumor, the present study aimed to analyze the nuclei and complete cell bodies of the tumor cells in different histopathological patterns of NLPHL and compare this to THRLBCL as a surrogate marker for genomic events taking place during the progression from typical NLPHL to variant patterns and THRLBCL.

## Materials and methods

### Samples

Written informed consent was obtained from all patients in accordance with the Declaration of Helsinki, and the study was approved by the Ethics Committee of the University Hospital Frankfurt (157/17).

In this study, cases of different NLPHL variant patterns according to Fan et al. and THRLBCL cases were analyzed in 2D and 3D. Diagnoses were confirmed by two expert hematopathologists (M.L.H. and S.H.).

### 2D imaging and image analysis

For this part of the project, 10 cases each of NLPHL patterns A, C, D, and E according to Fan et al.^7^ and 10 cases of THRLBCL were analyzed. 2D whole-tissue scans were acquired using a Panoramic Scan II (3D Histech) and the slides were digitalized with a 20x lens. For this step, all slides were stained with an anti-OCT2 antibody and the Envision Flex System was used for visualization. In order to analyze the scanned images, the open-source software QuPath (Version 0.1.2, 2014–2016; The Queen’s University of Belfast, Northern Ireland) was used^16^. For each case, 30 tumor cells (identified by the presence of large nuclei and strong OCT2 positivity) as well as 25 OCT2-negative lymphocytes, representing T cells, were manually encircled and the values of the area, perimeter, and diameter were acquired.

### Preparation of samples for 3D imaging

For 3D imaging, thick sections of up to 26 μm were cut from formalin-fixed, paraffin-embedded tissue as previously described^17,18^. Thick sections were deparaffinized according to standard protocols. For immunohistochemical double staining, dilutions of OCT2 (1:100, mouse monoclonal antibody, Cell Marque-Merck, Darmstadt, Germany/308M-18), CD20 (1:500, mouse monoclonal antibody, Agilent-Dako, Santa Clara, CA, USA, L-26), CD3 (1:200, rabbit polyclonal antibody, Agilent-Dako, A0452), PD1 (1:100, rabbit monoclonal, antibody, DCS, Hamburg, Germany, EP239), CD8 (mouse monoclonal antibody, 1:100, DAKO, M7103) and CD79a (1:100, rabbit monoclonal antibody, Cell Marque/179R-15 and 1:200, mouse monoclonal antibody, Agilent-Dako, M7050, Clone JCB117) were applied in a cocktail. For double stainings of T cells and LP cells either CD20 and CD3 were applied as double staining or PD1 and CD79a or CD8 and CD79a. As secondary antibodies, the VectaFluor Duet Immunofluorescence Double Labeling Kit DyLight 488/594 (Vector Laboratories, Burlingame, CA, USA, Cat. No. DK-8818) was used. In addition, nuclear DAPI staining (D9542; Sigma–Aldrich, St. Louis, USA) was performed.

### 3D imaging and image analysis

A Leica TCS SP8 confocal microscope (Leica Microsystems, Wetzlar, Germany) was used for 3D imaging. The settings were as follows: HC PL APO 63x/1.3 GLYC CORR, Cs2; lasers: 405 nm DMOD Compact, Red 594 nm and Green 488 nm. The presented pixel size was 130 nm in each coordinate direction. Furthermore, the z-step had a size of 0.13 mm^17,18^. For 3D image analysis, IMARIS Advanced Tracking 9.2 (Bitplane AG, Zurich, Switzerland) was utilized. First, surfaces of objects were calculated with the “Create Surface” tool using the default settings. Afterward, the “Source Channel” (green, red, and blue channel) was selected. “Absolute Intensity” was chosen for “Surface Creation Threshold”. The same steps were later carried out for the volume of the object. The surfaces and volumes of the OCT2^+^ nuclei of at least 25 tumor cells as well as their CD79a^+^ or CD20^+^ cell bodies were analyzed. Furthermore, the nuclei of 20 OCT2^+^ small B cells and 20 DAPI-stained, OCT2-negative, round lymphocyte nuclei corresponding to T cells were analyzed per case. T cells were additionally analyzed in CD3-, PD1- and CD8- immunostaining. All cells were identified based on morphology.

## Results

### Tumor cells of NLPHL and THRLBCL do not differ in nuclear perimeter and area in 2D histological sections

**Fig. 1:**
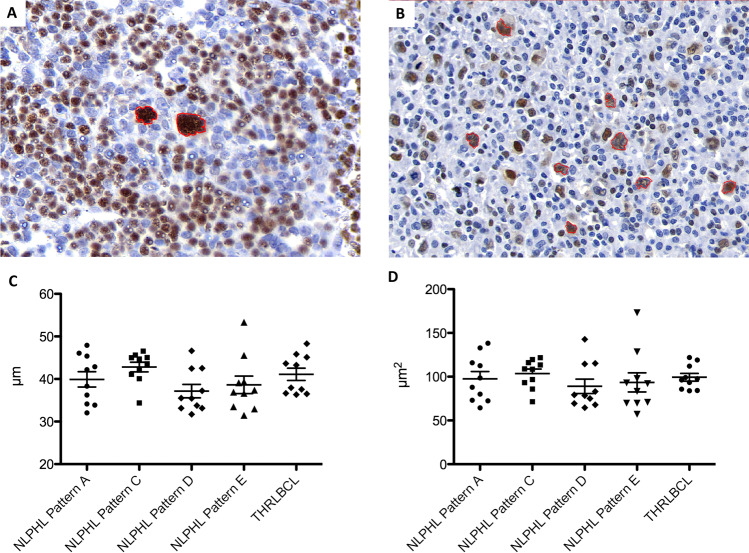
2D analysis of nuclear characteristics in different growth patterns of NLPHL and THRLBCL. **A** Example of 2D analysis of an NLPHL pattern A case showing LP cells with multilobated nuclei (OCT2-immunostaining, LP cells encircled in red). **B** Example of 2D analysis of a THRLBCL case showing tumor cells with angulated nuclei (OCT2-immunostaining, tumor cells encircled in red). **C** Nuclear perimeters of tumor cells from NLPHL patterns (A, C, D, and E) and THRLBCL in 2D analysis. **D** Nuclear areas of tumor cells from NHLPL patterns (A, C, D, and E) and THRLBCL in 2D analysis.

In 2D-scanned total slide sections, the area and perimeter of tumor cell nuclei from NLPHL pattern A, C, D, and E cases and THRLBCL were compared. The nuclear perimeter was slightly larger in the LP cells from NLPHL pattern C, indicating enhanced lobulation (Fig. 1). However, the difference was not significant. In order to rule out technical artifacts, we also compared the nuclear perimeters of the OCT2-negative T cells from the same scanned slides. However, no differences were seen here (Supplementary Fig. S1).

### 3D confocal imaging of tumor cells of NLPHL and THRLBCL reveals enlarged nuclear and cell sizes in THRLBCL

Since we did not see any obvious differences between different lymphoma types in 2D histological sections, we analyzed the 3D structure of the nuclei and cytoplasm of tumor cells from 10 cases each of different NLPHL patterns (A, C, and E according to Fan et al.^7^) and THRLBCL cases by confocal microscopy.

**Fig. 2:**
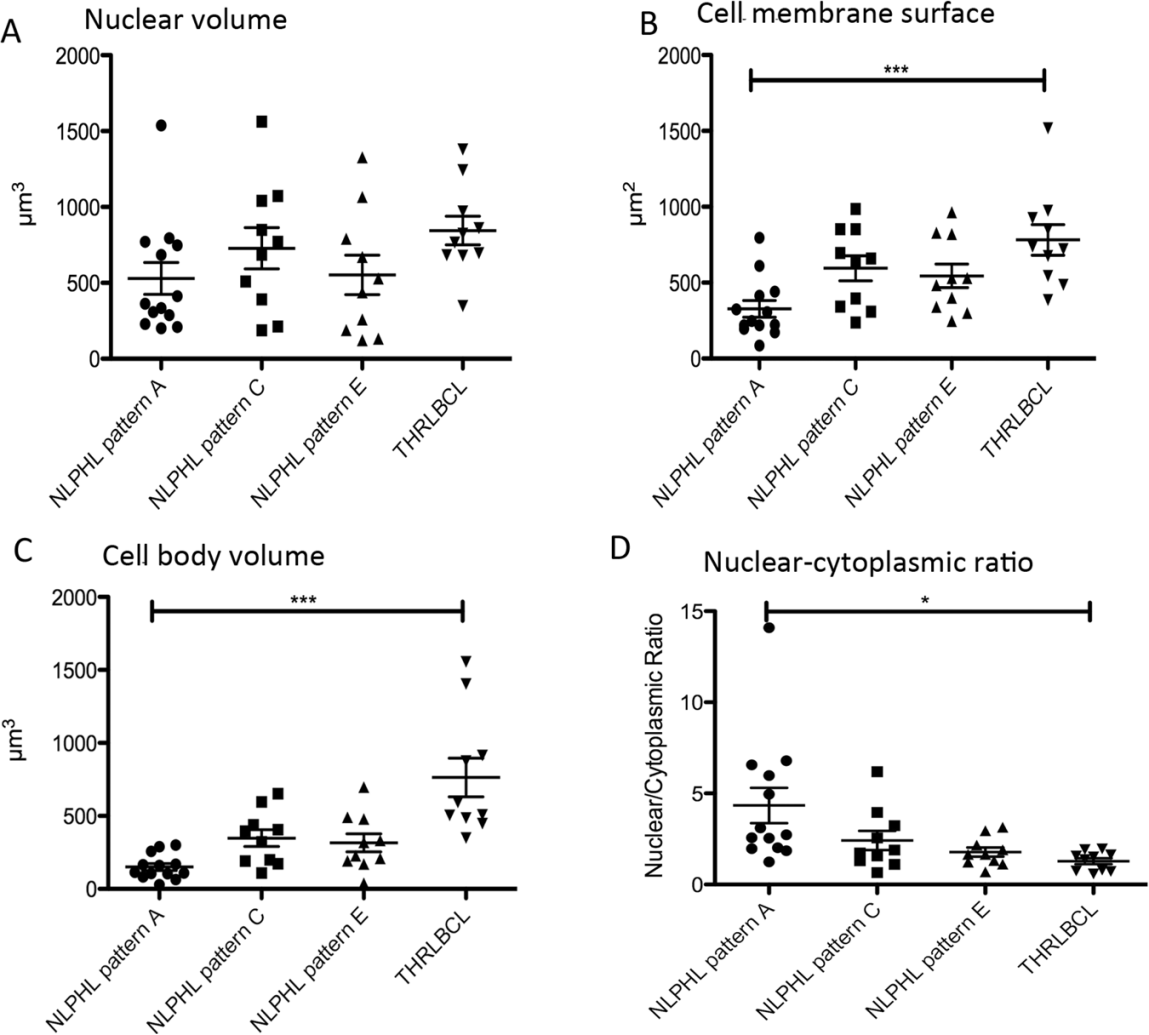
3D nuclear and cytoplasmic characteristics of the tumor cells of different NLPHL patterns and THRLBCL reveal an enlarged cell size of THRLBCL tumor cells. **A** Nuclear volumes of the tumor cells of NLPHL patterns (A, C, and E) and THRLBCL. **B** Cell membrane surfaces of the tumor cells of NLPHL patterns (A, C, and E) and THRLBCL (***p < 0.001, Kruskal–Wallis test with Dunn’s post hoc test for multiple comparisons). **C** Cell volumes of the tumor cells of NLPHL patterns (A, C, and E) and THRLBCL (***p < 0.001, Kruskal–Wallis test with Dunn’s post hoc test for multiple comparisons). **D** Ratio of nuclear/cytoplasmic volumes in NLPHL patterns (A, C, and E) and THRLBCL (*p < 0.05, Kruskal–Wallis test with Dunn’s post hoc test for multiple comparisons).

**Fig. 3:**
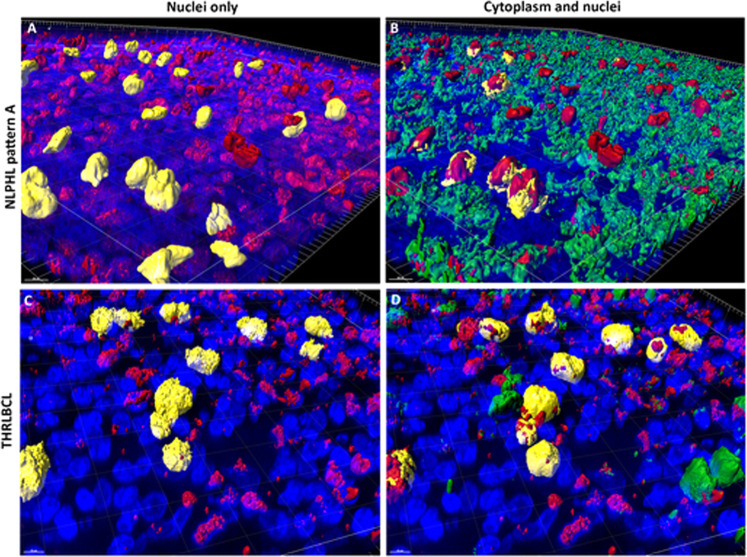
3D nuclear and cytoplasmic reconstruction of the tumor cells of exemplary NLPHL and THRLBCL cases. **A** 3D reconstruction of an OCT2 (red)-stained thick section of an NLPHL pattern A case. Selected LP cell nuclei are highlighted in yellow as examples of the multilobation of tumor cell nuclei. Nuclei are stained by DAPI (blue). **B** 3D reconstruction of an OCT2 (red) and CD79a (green) double-stained thick section of the NLPHL pattern A case. LP cell nuclei are labeled in red. CD79a^+^ cytoplasm is labeled in green. Cytoplasm of selected LP cells is highlighted in yellow, showing a scant cytoplasmic rim. Nuclei are stained by DAPI (blue). **C** 3D reconstruction of an OCT2 (red)-stained thick section of a THRLBCL case. Selected LP cell nuclei are highlighted in yellow and show examples of a spiculated tumor cell surface. Nuclei are stained by DAPI (blue). **D** 3D reconstruction of an OCT2 (red) and CD79a (green) double-stained thick section of the THRLBCL case. Tumor cell nuclei are labeled in red. CD79a^+^ cytoplasm is labeled in green. Cytoplasm of selected tumor cells is highlighted in yellow, showing a broader cytoplasmic rim. Nuclei are stained by DAPI (blue).

We analyzed both the surface of the nuclear membrane and the nuclear volume of the OCT2-labeled tumor cells of different variants of NLPHL as well as THRLBCL, since the LP cells of NLPHL are frequently multilobated, hence creating large nuclear surfaces at a moderate nuclear volume. We expected the tumor cells of THRLBCL to have a round shape at comparable volumes. 3D confocal microscopy revealed that the nuclear surfaces of the tumor cells of THRLBCL were comparable to the nuclear surfaces of NLPHL (means of 463–600 µm^2^ for NLPHL patterns A, C, and E and a mean of 639 µm^2^ for THRLBCL). However, at the same time, the nuclear volume of the tumor cells of THRLBCL was slightly increased (844 µm^3^ for THRLBCL vs. 528–728 µm^3^ for different NLPHL types), which fits well with the more roundish shape of the tumor cell nuclei in THRLBCL (Figs. 2A, 3).

We then analyzed the cytoplasm of the tumor cells, which was labeled by the intracellular CD79a antibody in a fluorescent double stain. Here, both the cell surface membrane and the cell volume were significantly increased in the tumor cells of THRLBCL when compared with typical NLPHL (cell surface THRLBCL mean 782 µm^2^ vs. NLPHL pattern A mean 327 µm^2^, *p* < 0.001, one-way-ANOVA with Bonferroni´s post hoc test for multiple testing; cell volume THRLBCL mean 763 µm^3^ vs. NLPHL pattern A mean 150 µm^3^, *p* < 0.001, Kruskal–Wallis test with Dunn’s post hoc test for multiple testing; (Figs. 2B, C and 3). Similar results were obtained when the tumor cell size was independently quantified in CD20 immunostaining (cell surface THRLBCL mean 1187 µm^2^ vs. NLPHL pattern A mean 555 µm^2^, *p* = 0.0017, unpaired t-test; cell volume THRLBCL mean 710 µm^3^ vs. NLPHL pattern A mean 303 µm^3^, *p* = 0.0007, unpaired *t* test; Supplementary Fig. S2). In order to exclude the effect of the nuclear cell volume on the total cell volume, we also studied the ratio of nuclear/cytoplasmic volume, which was significantly larger in typical NLPHL cases when compared with THRLBCL (4.34 vs. 1.82, *p* < 0.05, Kruskal–Wallis test with Dunn’s post hoc test for multiple testing; Fig. 2D).

### T cells in THRLBCL present significantly increased nuclear volumes when compared with NLPHL patterns A and C

**Fig. 4:**
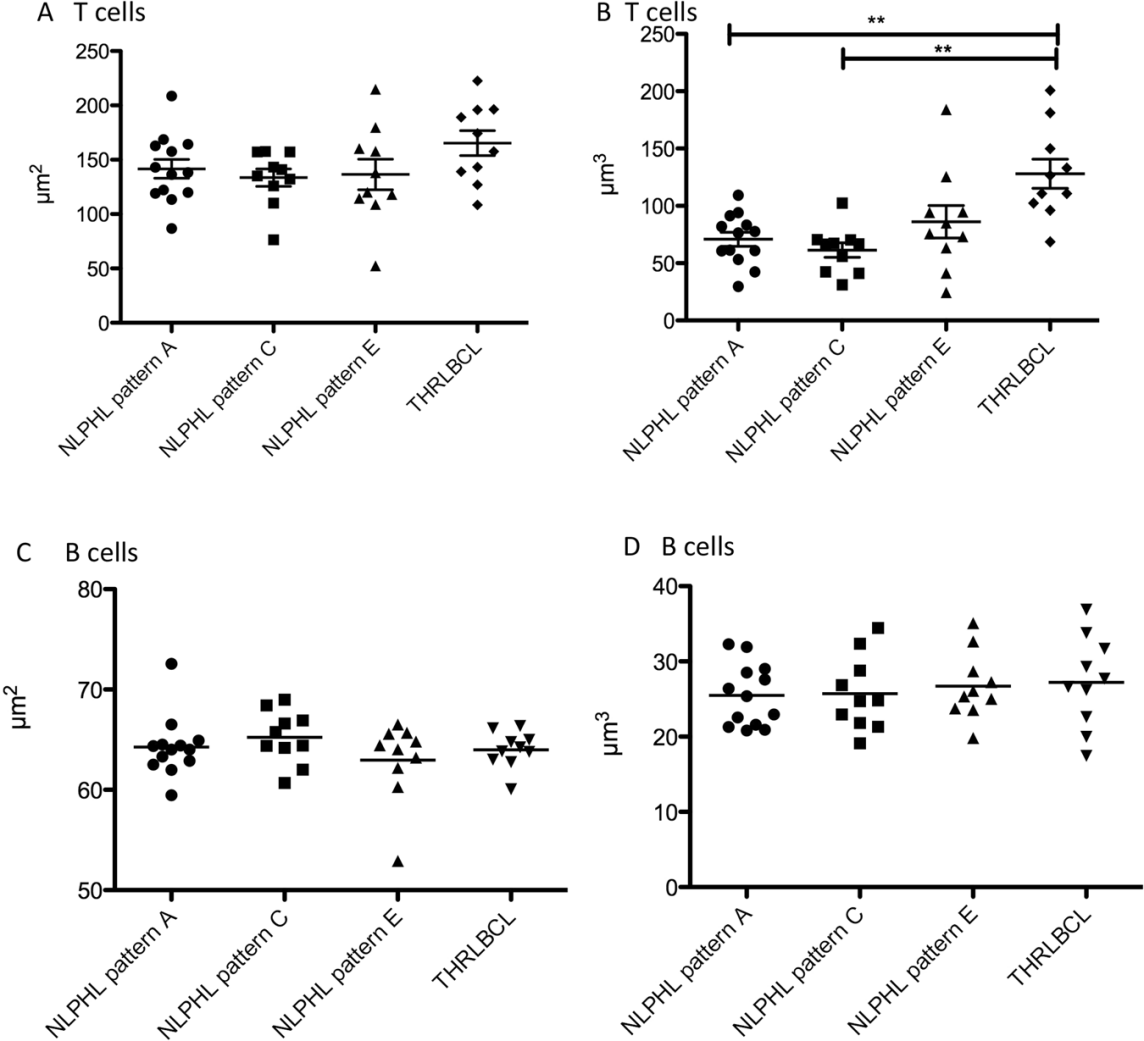
3D nuclear characteristics of the non-tumor cells of different NLPHL patterns and THRLBCL. **A** Surfaces of the DAPI-stained T cell nuclei of NLPHL patterns (A, C, and E) and THRLBCL. **B** Volumes of the DAPI-stained T cell nuclei are significantly enlarged in THRLBCL when compared with NLPHL patterns A and C (**p < 0.01, Kruskal–Wallis test with Dunn’s post hoc test for multiple comparisons). NLPHL pattern E T cells show a broad spectrum of volumes. **C** Surfaces of the OCT2-stained reactive B cell nuclei of NLPHL patterns (A, C, and E) and THRLBCL. **D** Volumes of the OCT2-stained reactive B cell nuclei of NLPHL patterns (A, C, and E) and THRLBCL.

**Fig. 5:**
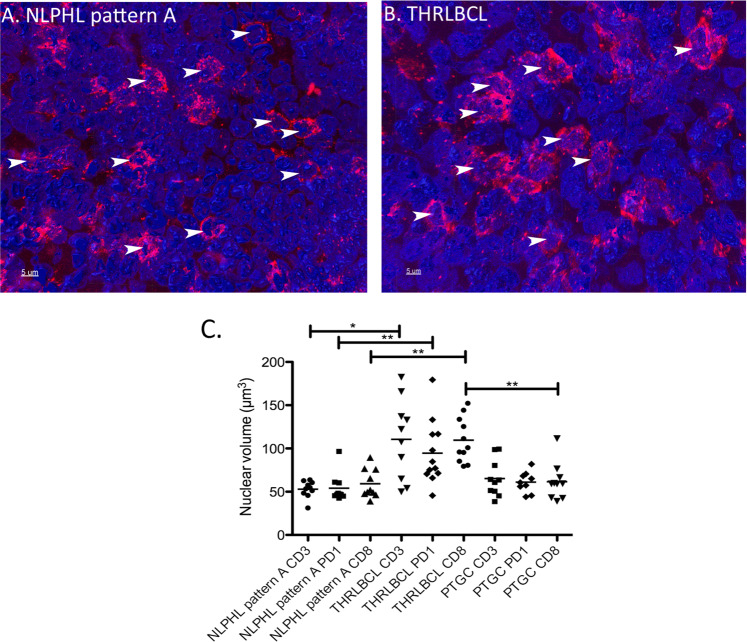
3D nuclear reconstruction of the T cells of an NLPHL pattern A case and THRLBCL. **A** 3D reconstruction of nuclei in a CD8-stained (red) thick section of an NLPHL pattern A case. Nuclei were counterstained with DAPI (blue). Selected nuclei of T cells are highlighted by arrows. 63x magnification. **B** 3D reconstruction of nuclei in a CD8-stained (red) thick section of a THRLBCL case. Nuclei were counterstained with DAPI (blue). Selected nuclei of T cells are highlighted by arrows. 63x magnification. **C** Nuclear volumes of CD3^+^, PD1^+^ and CD8^+^ T cells in NLPHL pattern A, THRLBCL and PTGC (*p < 0.05, **p < 0.01, Kruskal–Wallis-Test with Dunn’s post-test) quantified in 3D immunostained thick sections.

In addition to the analysis of tumor cells, nuclear volumes were measured for T cells (OCT2^−^, DAPI-stained lymphocyte nuclei) and reactive B cells (weakly OCT2^+^ nuclei). B cells served here as an internal control to rule out effects of processing and technical preparation. For a technical validation, nuclear sizes of OCT2^−^ lymphocytes (corresponding to T cells) and CD3^+^ lymphocytes (in separately stained thick sections) were compared in NLPHL pattern A and THRLBCL and similar results were obtained with both methods (Supplementary Fig. S3). The nuclear surfaces of the T cells were only slightly increased in the T cells from THRLBCL when compared with NLPHL (mean 194 µm^2^ in THRLBCL vs. means of 134–141 µm^2^ in NLPHL patterns A, C, and E, not significant; Fig. 4A). Surprisingly, the nuclear volumes of the T cells in THRLBCL were significantly enlarged (mean 137 µm^3^) when compared with the T-cell nuclei from NLPHL patterns A and C (means 71 and 61 µm^3^, respectively, *p* < 0.01, Kruskal–Wallis test with Dunn’s post hoc test for multiple comparisons; Figs. 4B, 5). In contrast, both the nuclear surfaces and nuclear volumes of normal B cells, serving as internal control, did not vary among the different NLPHL patterns A, C, and E and THRLBCL (Fig. 4C, D).

In order to elucidate if the observed nuclear size changes reflect a different composition of the microenvironment, T-cell nuclei of PD1^+^ and CD8^+^ T cells were analyzed separately and compared. All T-cell subtypes in THRLBCL had significantly larger nuclear volumes (means of 123, 110 and 111 µm^3^ for CD3^+^, CD8^+^ and PD1^+^ T cells) when compared with NLPHL pattern A (means of 53, 59 and 54 µm^3^ for CD3^+^, CD8^+^ and PD1^+^ T cells, *p* < 0.05, Kruskal–Wallis-Test with Dunn´s post-test for multiple testing, Fig. 5). Progressively transformed germinal centers (PTGC), a reactive lesion with a strong morphologic similarity to NLPHL, presented T cells with nuclei similar to NLPHL (means of 65, 61 and 61 µm^3^ for CD3^+^, CD8^+^ and PD1^+^ T cells). There were no significant differences between PD1^+^ and CD8^+^ T cells within one category investigated (Fig. 5).

### Tumor cells of THRLBCL are in close contact with CD8^+^ T cells

**Fig. 6:**
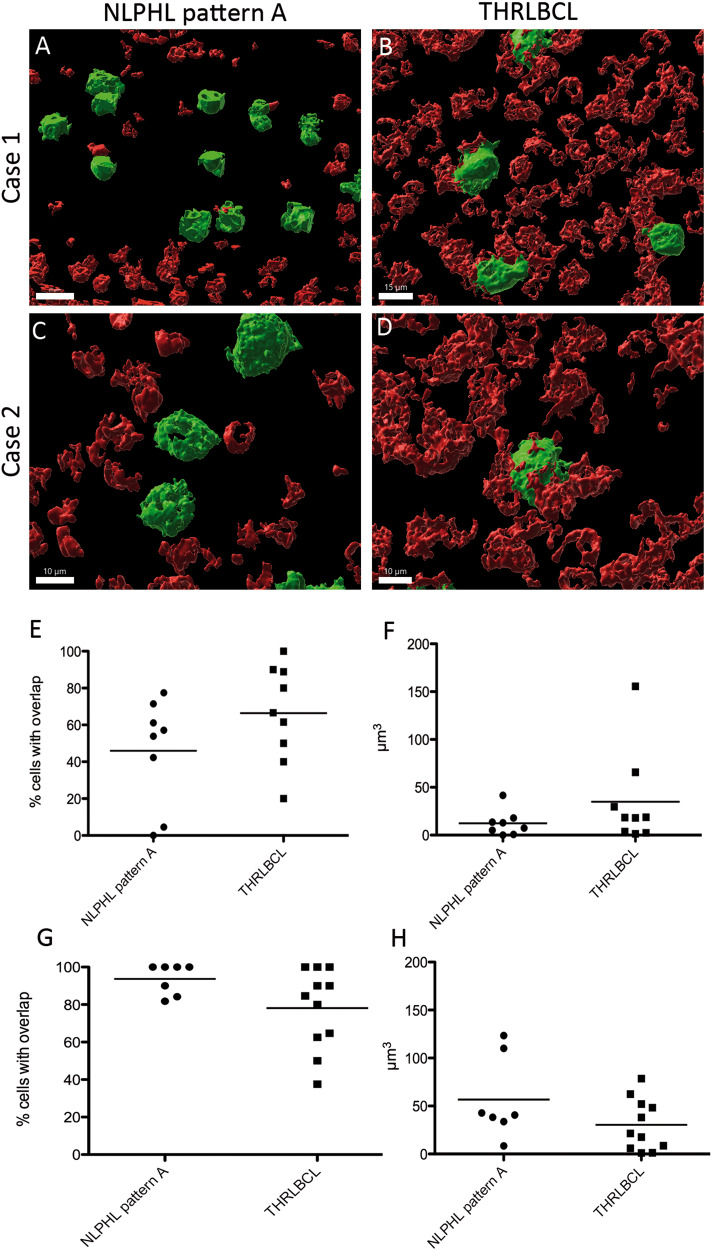
Tumor cells in THRLBCL have more contacts with CD8^+^ T cells than LP cells in NLPHL pattern A. **A**–**D** 3D reconstruction of thick sections double-stained with CD8 and CD79a. Tumor cells were identified by the large cell size and positivity for CD79a. **A**, **C** Examples from two NLPHL pattern A cases show little contact faces of tumor cells with CD8^+^ cells. **B**, **D** Examples from two cases of THRLBCL present many and relatively large overlapping surfaces between tumor cells and CD8^+^ T cells. **E** Mean percentage of tumor cells with contact to CD8^+^ T cells in NLPHL and THRLBCL (Mann–Whitney-test, not significant). **F** Mean volumes of contacts between CD8^+^ T cells and tumor cells in NLPHL and THRLBCL (Mann–Whitney-test, not significant). **G** Mean percentage of tumor cells with contact to PD1^+^ T cells in NLPHL and THRLBCL (Mann–Whitney-test, not significant). **H** Mean volumes of contacts between PD1^+^ T cells and tumor cells in NLPHL and THRLBCL (Mann–Whitney-test, not significant).

We also studied the cellular interactions between tumor cells of NLPHL pattern A and THRLBCL on the one hand and CD8^+^ and PD1^+^ T cells in 3D thick tissue slices double-stained with CD79a on the other hand. The percentage of tumor cells that had direct contact with CD8^+^ T cells was slightly higher in THRLBCL when compared with NLPHL pattern A (66% vs. 46% in THRLBCL vs. NLPHL pattern A, not significant, Fig. 6A–E). The same tendency was observed when the overlapping faces between cell membranes of CD8^+^ cells and neoplastic cells were measured (35 vs. 12 µm^3^ in THRLBCL vs. NLPHL pattern A, not significant, Fig. 6B, D, F). In contrast, LP cells of NLPHL had more frequent contacts with PD1^+^ T cells and slightly larger overlapping faces with PD1^+^ T cells when compared with tumor cells from THRLBCL (57 vs. 30 µm^3^, not significant, Fig. 6G, H).

## Discussion

This paper focuses on the nuclear characteristics and cell sizes in NLPHL and THRLBCL, considering different histological NLPHL variant growth patterns. In previous studies^14,15,19^, the hypothesis that NLPHL variant patterns represent progression forms of these lymphomas whereas THRLBCL represents transformation into an aggressive lymphoma was generated. Since the aggressiveness of tumors correlates with nuclear atypia, we assessed the nuclear morphology of tumor and bystander cells as well as tumor cell shape. The architecture of cancer cell nuclei is often diagnostic, as are the usually observed popcorn-like nuclei of LP cells in NLPHL^20^. However, factors leading to such characteristic nuclear morphological features are multifactorial and include differences in the protein composition of the nuclear matrix, differences in chromatin texture related to the activation of different parts of the genome, and differences in the number and structural organization of chromosomes^21^. In 2D histological sections, no apparent difference between the tumor cell nuclei of NLPHL variants and THRLBCL was observed. In contrast, in 3D confocal laser images, there were slight differences in nuclear size and more importantly, differences in the cell body surface and volume. 3D analysis of cell sizes is considered more powerful since in 3D, a 10% increase in cell diameter results in a 33% increase in volume^22^. An increase in tumor cell volume was observed in the NLPHL variant patterns C and E; suggesting a step-by-step progression. However, the difference in tumor cell volume was significant only in THRLBCL when compared with the typical NLPHL pattern A. Surprisingly, the nuclear volume of tumor cells was only slightly increased in the NLPHL variant patterns and THRLBCL. Also of interest, the nuclear/cytoplasmic ratio was largest in the typical NLPHL pattern A and smallest in the tumor cells of THRLBCL. This contrasts with expected results. The size and shape of tumor nuclei can reflect genomic instability on the one hand and are also related to different functional states on the other. A lower nuclear/cytoplasmic ratio in THRLBCL could be explained, for example, by higher metabolic and transcriptional activity. Another hypothesis may be the influence of follicular dendritic cells (FDCs) on the tumor cells in NLPHL. FDCs are absent in THRLBCL^7,23^ A pro-proliferative effect and protection from apoptosis was shown for B cells that have intimate contacts with FDCs^24–26^. Thus, the interaction of tumors cells with FDCs might have an additional effect on nuclear and cytoplasmic volume of the tumor cells. Another alternative hypothesis may be increased cell swelling after cell damage as a possible explanation, leading to enhanced cytoplasmic volume^22,27^. A larger and hydropic cytoplasmic volume can be seen in cells that have experienced cellular stress^28,29^. The fact that T cells in THRLBCL, in particular, had significant differences in nuclear volume suggests that the functional state and activation of T cells differs in THRLBCL from NLPHL. Surprisingly, not only CD8^+^ T cells, but T cells in general (CD3, PD1) presented enlarged nuclei in THRLBCL, pointing to a completely different immune microenvironment activation situation. It is known that activation of T cells leads to an increased size^30^ and also to enlarged and elongated nuclei^31,32^, suggesting an activation of the T cells in THRLBCL. The fact that CD8^+^ T cells were in close vicinity to tumor cells of THRLBCL with many overlapping cell surfaces supports the hypothesis of cellular stress of the tumor cells in THRLBCL leading to an increased cell body volume (Fig. 3D, Supplementary Fig. S4). Tumor cells and surrounding macrophages in THRLBCL frequently express PD-L1^33^ possibly rendering CD8^+^ cells into an unresponsive state so that the tumor remains in a balance between cytotoxic host response and tumor survival via inhibition of effector functions of CD8^+^ cells.

Differences in the composition of the microenvironment of NLPHL and THRLBCL have previously been observed^34,35^, with a higher number of macrophages and CD8^+^ T cells in THRLBCL. Similarly, Boudova et al.^36^ found a higher number of activated cytotoxic T cells in the microenvironment of THRLBCL. In the present study we did not quantify the number of CD8^+^ T cells, since they were frequently numerous with confluent cell bodies in THRLBCL. However, in a previous study we observed a gradual increase with a strong individual variability in CD8^+^ cells from NLPHL variants towards THRLBCL and also increased CD8^+^ counts in diffuse large B-cell lymphomas transformed from NLPHL^19,37^.

In summary, we see differences in the tumor cell size and nuclear size of reactive T cells in THRLBCL when compared with the typical histopathologic growth pattern of NLPHL. Despite the fact that the size and shape of cells remain descriptive, our results are in line with previous data^34^, emphasizing that the microenvironment is the largest discriminating factor between typical NLPHL and THRLBCL, with an activated CD8^+^ T cell population in THRLBCL closely attached to tumor cells of THRLBCL leading to cellular stress of the tumor cells, which may explain the low tumor cell content as a result of ineffective proliferation. Furthermore, we observe in the present study gradual changes from NLPHL pattern A over the atypical patterns with a maximum in THRLBCL, suggesting a step-wise progression and transformation process. This may be the reason why the histopathological delineation of THRLBCL from NLPHL pattern E is frequently difficult, particularly in small biopsies.

## Supplementary information

Supplementary Figures

## Data Availability

Original data represent large files of 3D images that are available on request from the corresponding author.
