# Supplementary material for: 3D analyses reveal T cells with activated nuclear features in T-cell/histiocyte-rich large B-cell lymphoma
Source: Mod Pathol. 2022 Feb 16;35(10):1431–8. doi: 10.1038/s41379-022-01016-8 (PMC9514992; doi:10.1038/s41379-022-01016-8)
Supplement: Supplementary file 1 — Supplementary Figures [file 41379_2022_1016_MOESM1_ESM.pdf]

Supplementary Figures

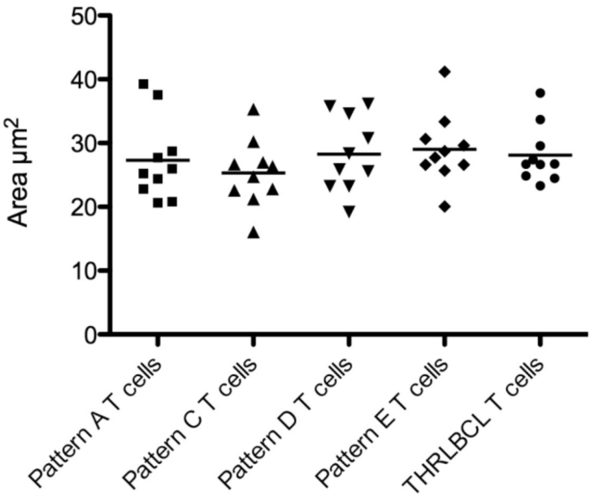

Supplementary Figure S1. Quantification of nuclear area of reactive T cells (OCT2-negative lymphocytes) in different growth patterns (A, C, D and E) of NLPHL and THRLBCL in 2D.

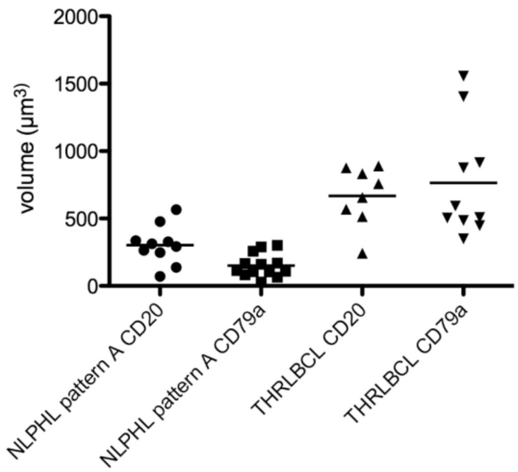

Supplementary Figure S2. Quantification of the 3D volume of the tumor cell body in NLPHL pattern A and THRLBCL. Tumor cell volumes were independently quantified in CD20 and CD79a immunostainings yielding comparable results.

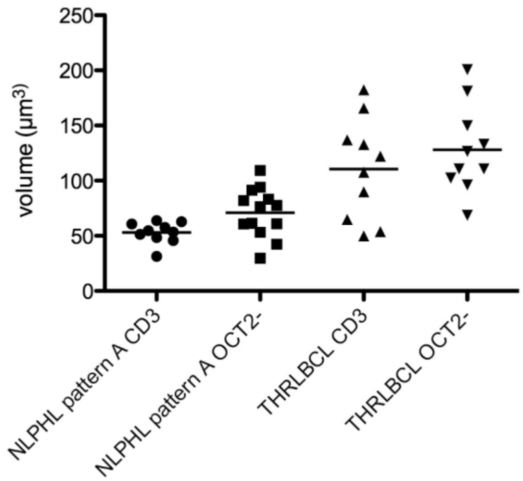

Supplementary Figure S3. Quantification of the 3D nuclear volume of T lymphocytes in NLPHL pattern A and THRLBCL. T lymphocyte nuclear volumes were independently quantified in either OCT2 immunostaining, chosing lymphocytes with OCT2-negative nuclei, or in CD3-immunostaining, chosing positively labelled cells.

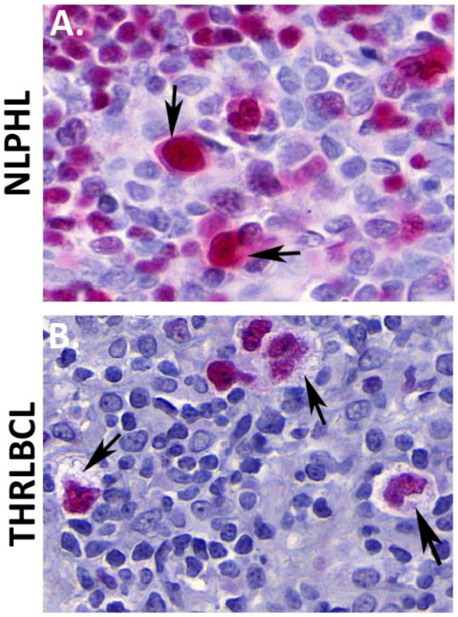

Supplementary Figure S4. Examples of 2D histological slides of NLPHL pattern A and THRLBCL with OCT2 staining (40x magnification).

A. Examples of LP cells in NLPHL pattern A with a scant, moderately $OCT2^+$ cytoplasm (highlighted by arrows).
B. Examples of tumor cells of THRLBCL with a broad and pale, vacuolated cytoplasm hinting at cellular swelling (highlighted by arrows).
